# Eating habit of adding salt to foods and incident sleep apnea: a prospective cohort study

**DOI:** 10.1186/s12931-022-02300-6

**Published:** 2023-01-07

**Authors:** Tingting Li, Lin Song, Guang Li, Fengping Li, Xiaoge Wang, Liangkai Chen, Shuang Rong, Li Zhang

**Affiliations:** 1 Academy of Nutrition and Health, Hubei Province Key Laboratory of Occupational Hazard Identification and Control, School of Public Health, Wuhan University of Science and Technology, Wuhan, 430065 China; 2 Department of Neurology, Hubei Provincial Hospital of Integrated Chinese and Western Medicine, Xinhua Hospital of Hubei University of Chinese and Western Medicine, Wuhan, 430015 China; 3 Department of Epidemiology, School of Public Health, Wuhan University, Wuhan, 430062 China; 4 Department of Nutrition and Food Hygiene, Hubei Key Laboratory of Food Nutrition and Safety, Ministry of Education Key Lab of Environment and Health, School of Public Health, Tongji Medical College, Huazhong University of Science and Technology, Wuhan, 430030 China

**Keywords:** Adding salt, Sleep apnea, Salt reduction program

## Abstract

**Background:**

Previous studies have revealed that sodium-restricted diet intervention significantly decreased apnea frequency among patients with sleep apnea. However, the longitudinal association between the habit of adding salt to foods and sleep apnea in general populations is uncertain.

**Methods:**

The UK Biobank cohort study includes more than 500,000 participants aged 40 to 69 across the United Kingdom from 2006 to 2010. The frequency of adding salt to foods was collected through a touch screen questionnaire. Incident sleep apnea was ascertained by hospital inpatient records, death registries, primary care, and self-reported diagnosis. The association between the habit of adding salt to foods and incident sleep apnea was estimated using Cox proportional hazard regression models.

**Results:**

Among the 488,196 participants (mean age 56.5 years; 55.0% female) in this study. During a median follow-up of 12.3 years, 6394 sleep apnea events occurred. Compared to participants who never/rarely added salt to foods, those who sometimes, usually, and always added salt to foods had an 11% (hazard ratio [HR] 1.11, 95% confidence interval [CI]: 1.04 to 1.17), 15% (HR 1.15, 95% CI: 1.07 to 1.24), and 24% (HR 1.24, 95% CI: 1.12 to 1.37) higher risk for incident sleep apnea, respectively.

**Conclusions:**

In this large prospective study, the habit of adding salt to foods was associated with a higher risk of incident sleep apnea. The findings support the benefits of a salt reduction program in preventing sleep apnea.

**Supplementary Information:**

The online version contains supplementary material available at 10.1186/s12931-022-02300-6.

## Introduction/background

Sleep apnea is a common sleep-disordered breathing condition that affects an estimated 23% of women and 50% of men aged 40–85 in the general population [1]. Sleep apnea has a major impact on morbidity and mortality and has been recognized as an independent risk factor for motor vehicle crashes and occupational accidents [2–4], heart failure [5, 6], stroke [7, 8], atrial fibrillation [9, 10], and cognitive impairment [11]. Given the global economic and medical burden it posed [12], cost-effective public health interventions should be encouraged to curb the sleep apnea epidemic through modifiable risk factors.

High sodium/salt intake is one of the leading contributors to disease burden across the world [13]. High sodium intake may also play a role in the pathogenesis of sleep apnea through the mechanism of fluid retention [14]. The preliminary evidence from a clinical trial showed that a sodium-restricted diet intervention promoted a significant decrease of apnea frequency among men with severe obstructive sleep apnea [15, 16]. Major dietary sources of sodium are from salt added during the processing and manufacture of foods (non-discretionary) and salt added to food during cooking and to foods (discretionary) [17]. A nationally representative survey reported that 32.5% adults generally added salt to foods in England [18]. Moreover, it was estimated that 15–20% of salt in the diet obtained from salt adding to foods [17]. However, it remains uncertain whether the habit of adding salt to foods is associated with the risk of sleep apnea. We aimed to prospectively examine the above association in a large prospective cohort from UK Biobank. This evidence may contribute to public health policy, education, and promotion.

## Methods

### Study population

UK Biobank recruited over 500,000 participants aged 40–69 between 2006 and 2010 [19]. Participants attended 1 of the 22 assessment centers across England, Wales, and Scotland, where they completed a touch-screen questionnaire, had physical measurements taken, and provided biological samples, as described in detail elsewhere [20]. Health-related outcomes of the participants were collected through linkages to electronic health records, including inpatient hospital records, primary care records, and death registrations. UK Biobank has ethics approval from the North Multi-centre Research Ethics Committee (11/NW/0382). All participants provided informed written consent. This research has been conducted using the UK Biobank Resource under project numbers 63454 and 69,424.

**Fig. 1:**
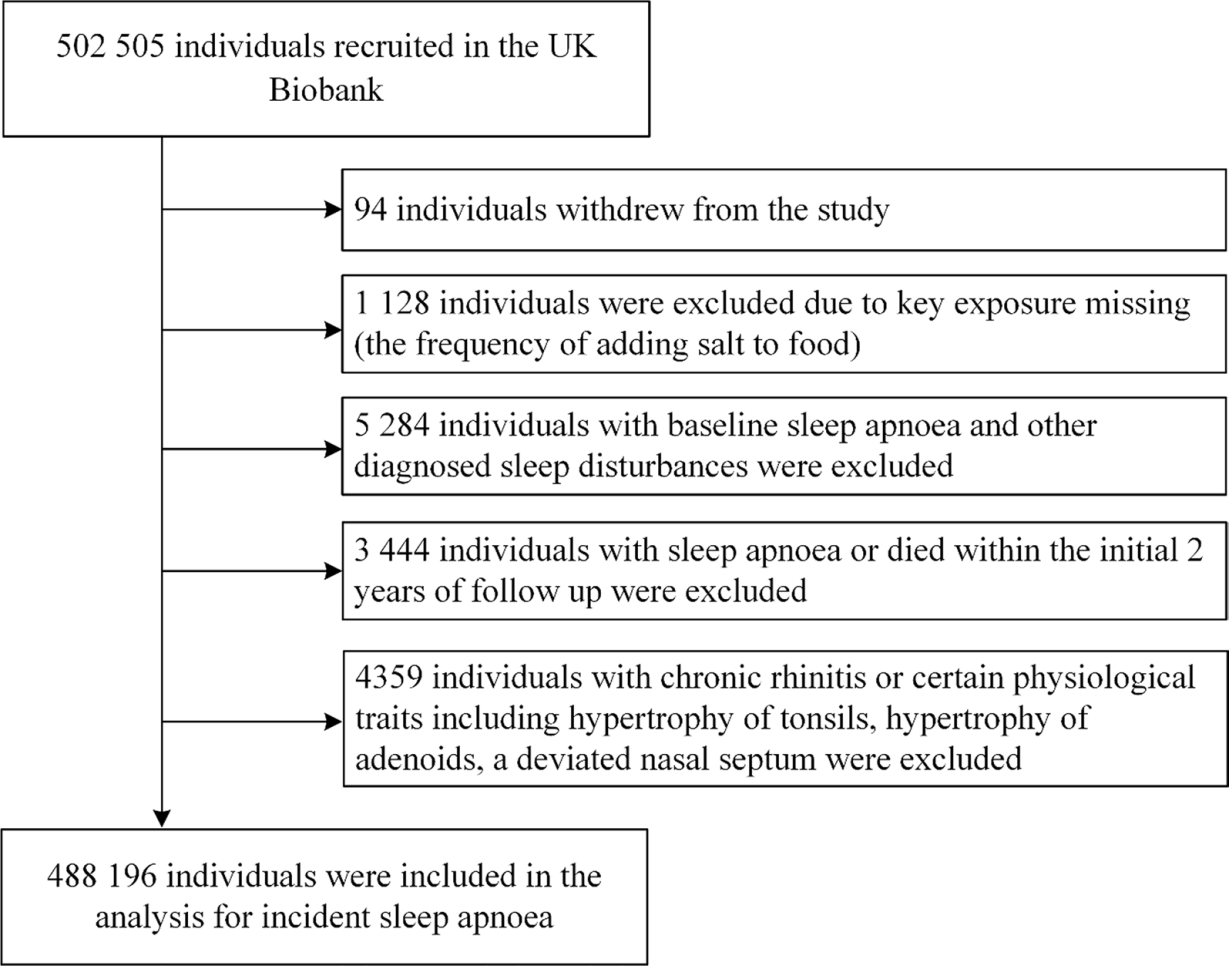
Flowchart of participant selection

We excluded participants who withdrew (n = 94), those with incomplete data on the frequency of adding salt to foods (n = 1128), participants with sleep apnea or other diagnosed sleep disturbances at baseline (n = 5284), and participants with sleep apnea or died within the initial 2 years of follow up (n = 3444). Our final analysis included 488,196 participants (Fig. 1).

### Exposure assessment

Participants attended 1 of 22 assessment centers across the UK, where they completed a touch screen questionnaire. One of the questions asked, “Do you add salt to your food? (Do not include salt used in cooking)”, and participants could select one answer from the given options: “Never/rarely”, “Sometimes”, “Usually”, “Always”, and “Prefer not to answer”. In the primary analysis, salt use was coded as an ordered categorical variable with four levels of discretionary salt use.

### Ascertainment of outcomes

Prevalent and incident sleep apnea cases within the UK Biobank were ascertained through data linkage to hospital inpatient records, death registries, primary care, and self-reported fields. The date of diagnosis was set as the earliest date of sleep apnea codes recorded regardless of the source used. According to the International Classification of Disease (ICD) edition 10, sleep apnea was defined as code G47.3. Participants’ self-reported diagnosis was identified from self-reported non-cancer illness code (1123) in the data field 20,002 (Additional file 1: Table S1).

### Covariates

Potential confounders were acquired using a baseline touch-screen questionnaire, including age, sex, ethnicity, household income, highest qualification, employment status, smoking status, and alcohol intake. The Townsend deprivation index was applied to indicate socioeconomic status, and a high score was equated with a high socioeconomic deprivation [21]. In accordance with the healthy physical activity recommendations [22], we defined physical activity as inactive (no documented moderate or vigorous physical activity), insufficient (moderate activity of < 150 min/week and vigorous activity of < 75 min/week), and active (moderate activity of ≥ 150 min/week or vigorous activity of ≥ 75 min/week or equivalent). The mean values of vegetable intake, fruit intake, processed meat intake, and unprocessed red meat intake were used in this study. BMI was calculated by dividing a participant’s weight, measured to the nearest 0.1 kg using the Tanita BC-418 MA body composition analyzer (Tanita Corporation of America, IL), by the square of their standing height in meters, measured with a Seca 202 device (SECA, Hamburg, Germany). Hypertension, diabetes, atrial fibrillation, congestive heart failure, stroke, and asthma at baseline were ascertained using ICD diagnosis codes, record-linkage data from local general practitioners, or self-reported previous diagnoses. Menopause and history of hormone replacement therapy were self-reported. A casual (spot) urine was obtained at the end of about a 2-h visit and stored at -80℃. Sodium and potassium concentrations were measured at the UK Biobank laboratory from stored urine aliquots by the Ion Selective Electrode (potentiometric) method using Beckman Coulter AU5400. We estimated 24-h sodium excretion and 24-h potassium excretion (mg/day) from the spot urinary concentration values based on the Kawasaki formula [23]. Information on sleep duration, chronotype, and major dietary changes in the last 5 years were acquired using baseline touch-screen questionnaire. Drug use, including depressants and drugs for blood pressure and blood sugar, was ascertained by a touch-screen questionnaire and verbal interview. More details are shown in Additional file 1: Table S2.

### Statistical analysis

Descriptive characteristics of the sample are presented as either mean (SD) or number (percentage). Follow-up time was calculated from the baseline date to diagnosis of the outcome, death, or the censoring date (30 September 2021 in England, 31 July 2021 in Scotland, and 28 February 2018 in Wales), whichever occurred first. The sleep apnea incidence rate (per 1000 person-years) was calculated, and the Poisson regression model was assumed to calculate the confidence intervals (CIs). Cox proportional hazards regression models were used to estimate the hazard ratio (HR) and 95% CI. The proportional hazard assumption was evaluated by tests based on Schoenfeld residuals [24], and no violation of this assumption was observed. We ran four incremental models for outcome: ‘model 1’ included age, sex, race (White and non-White); ‘model 2’ additionally included sociodemographic covariates: educational attainment (college or university, vocational, upper secondary, lower secondary, or others), socioeconomic status (quintiles of Townsend deprivation index), household income (< £18 000, £18 000-£30 999, £31 000-£51 999, £52 000-£100 000, > £100 000, or unknown), employment status (employed, unemployed, or retired), and 22 assessment centers; ‘model 3’ additionally included lifestyle factors: smoking status (never, previous, current, or unknown), alcohol intake (moderate alcohol intake [yes/no]), physical activity (inactive, insufficient, or recommended level), vegetable intake (quartiles), fruit intake (quartiles), processed meat intake (quartiles), unprocessed red meat intake (quartiles), BMI (< 18.5 kg/m^2^, 18.5 ~ 25 kg/m^2^, 25 ~ 30 kg/m^2^, ≥ 30 kg/m^2^); ‘model 4’ additionally included multimorbidity: diabetes (yes/no), hypertension (yes/no), atrial fibrillation (yes/no), congestive heart failure (yes/no), stroke (yes/no), and asthma (yes/no). If covariate information was missing, we used a missing indicator approach for categorical variables and with mean values for continuous variables [25].

To assess the potential effect modification, stratified analyses, and interaction analyses were performed by the following factors: sex (women or men), age (< 60 or ≥ 60), race (White, non-White), socioeconomic status (dimidiate Townsend deprivation index), physical activity (active, insufficient, and inactive), smoking status (never, ever, and current), moderate drinking (yes, no), BMI (< 25, 25.0–29.9, or ≥ 30), hypertension (yes, no), diabetes (yes, no), atrial fibrillation (yes, no), congestive heart failure (yes, no), stroke (yes, no), and asthma (yes, no).

We conducted several sensitivity analyses. First, to examine robustness against missing covariates, we repeated the main analysis using a complete analysis approach. Second, to examine robustness against severe medical conditions, we repeated the main analysis by excluding those with CVD or cancer at baseline. Third, to assess the risk of the habit of adding salt to foods with the outcome, salt use was coded into a binary variable (“generally add salt to foods” and “do not generally add salt to foods”) as an independent variable in Cox models. Fourth, to examine robustness against great changes in eating habits, we repeated the main analysis by excluding those who reported great changes in eating habits. Fifth, we repeated the main analysis in participants with low OSA risk to minimize the undiagnosed cases of sleep apnea. Based on Berlin Questionnaire, the risk of OSA was determined by reports of snoring, daytime sleepiness, body mass index (BMI), and blood pressure [26]. Sixth, the hormonal status might be related to the prevalence of sleep-disordered breathing in females [27, 28]. We further adjusted for menopause and history of hormone replacement therapy among females. Seventh, we adjusted for other potential covariates, including sodium and potassium in the urine, sleep duration, chronotype, weight change compared with 1 year ago, waist circumference, depressant use, drugs for blood pressure and blood sugar, and diuretics. Eighth, we evaluated the potential mediation effect by modeling the cross-product term of the blood pressure, BMI, and C-reactive protein level with the frequency of salt added to foods [29].

All statistical analyses were conducted using SAS version 9.4 (SAS Institute, Cary, NC), and we considered a two-sided *P* value less than 0.05 statistically significant.

## Results

The baseline characteristics of the study participants according to the frequency of adding salt to foods are in Table 1. Among 488,196 participants 38 to 73 years of age (mean age 56.5 ± 8.1 years, 55.0% female) in this study, 55.6% (n = 271,311) never or rarely added salt to foods, 28.0% (n = 136,763) sometimes added salt to foods, 11.6% (n = 56,544) usually added salt to foods, and 4.8% (n = 23,578) always added salt to foods at baseline. Compared with participants who never/rarely added salt to foods, those who usually or always added salt to foods were heavier, likely to be men, non-white, unemployed, inactive, current smokers, non-moderate drinkers, and with less household income, lower education level, more urinary sodium excretion, less urinary potassium excretion, and had a higher prevalence of diabetes, congestive heart failure, stroke, and asthma.

**Table 1:** Baseline characteristics of 488,196 participants stratified by the frequency of salt added to food in UK Biobank study

Baseline characteristics	Salt added to food			
	Never/rarely (n = 271,311)	Sometimes (n = 136,763)	Usually (n = 56,544)	Always (n = 23,578)
Age, mean (SD), years	56.52 (8.08)	56.41 (8.12)	56.98 (8.04)	55.91 (8.27)
Female sex, n (%)	153,415 (56.55)	74,648 (54.58)	27,939 (49.41)	12,293 (52.14)
Whites, n (%)	259,100 (95.5)	127,655 (93.34)	52,824 (93.42)	20,646 (87.56)
Townsend deprivation index, median (IQR)	− 2.32 (− 3.73–0.18)	− 2.05 (− 3.59–0.66)	− 1.93 (− 3.54–0.9)	− 0.93 (− 3.07–2.47)
*BMI, mean (SD), kg/m*^*2*^	27.12 (4.68)	27.58 (4.77)	27.76 (4.76)	27.97 (5.05)
< 18.5	1507 (0.56)	616 (0.45)	265 (0.47)	169 (0.72)
18.5–24.9	94,606 (34.87)	42,201 (30.86)	16,203 (28.66)	6632 (28.13)
25.0–29.9	113,666 (41.9)	58,687 (42.91)	24,661 (43.61)	9830 (41.69)
≥ 30.0	60,281 (22.22)	34,522 (25.24)	15,036 (26.59)	6738 (28.58)
Missing	1251 (0.46)	737 (0.54)	379 (0.67)	209 (0.89)
*Education, n (%)*				
College or university	93,358 (34.41)	42,573 (31.13)	16,934 (29.95)	4750 (20.15)
Vocational	30,502 (11.24)	16,279 (11.9)	6993 (12.37)	2989 (12.68)
Upper secondary	31,705 (11.69)	14,642 (10.71)	5830 (10.31)	1857 (7.88)
Lower secondary	70,712 (26.06)	36,561 (26.73)	15,003 (26.53)	6327 (26.83)
Others	40,560 (14.95)	24,011 (17.56)	10,699 (18.92)	7018 (29.77)
Missing	4474 (1.65)	2697 (1.97)	1085 (1.92)	637 (2.7)
*Household income, £*				
< 18,000	49,007 (18.06)	26,554 (19.42)	11,792 (20.85)	6506 (27.59)
18,000–30,999	58,269 (21.48)	29,497 (21.57)	12,453 (22.02)	4973 (21.09)
31,000–51,999	61,426 (22.64)	30,357 (22.2)	12,114 (21.42)	4204 (17.83)
52,000–100,000	49,433 (18.22)	23,056 (16.86)	9191 (16.25)	2750 (11.66)
> 100,000	13,282 (4.9)	6165 (4.51)	2486 (4.4)	622 (2.64)
Missing	39,894 (14.7)	21,134 (15.45)	8508 (15.05)	4523 (19.18)
*Employment, n (%)*				
Employed	157,089 (57.9)	79,475 (58.11)	31,419 (55.57)	12,443 (52.77)
Unemployed	20,491 (7.55)	11,499 (8.41)	5179 (9.16)	3578 (15.18)
Retired	91,398 (33.69)	44,452 (32.5)	19,365 (34.25)	7192 (30.5)
Missing	2333 (0.86)	1337 (0.98)	581 (1.03)	365 (1.55)
*Physical activity, n (%)*				
Recommended level	140,265 (51.7)	68,667 (50.21)	27,729 (49.04)	10,689 (45.33)
Insufficient	75,229 (27.73)	37,798 (27.64)	15,326 (27.1)	5619 (23.83)
Inactive	39,995 (14.74)	20,768 (15.19)	9600 (16.98)	4970 (21.08)
Missing	15,822 (5.83)	9530 (6.97)	3889 (6.88)	2300 (9.75)
Current smoking, n (%)	21,571 (7.95)	15,362 (11.23)	8566 (15.15)	5537 (23.48)
Moderate drinking, n (%)	146,975 (54.17)	72,109 (52.73)	27,901 (49.34)	9804 (41.58)
Vegetables (SVs/d), mean (SD)	2.53 (1.71)	2.33 (1.67)	2.15 (1.71)	1.95 (1.86)
Fruits (SVs/d), mean (SD)	1.65 (1.09)	1.61 (1.11)	1.61 (1.16)	1.58 (1.34)
Red meat (SVs/d), mean (SD)	2.01 (1.39)	2.17 (1.45)	2.3 (1.54)	2.43 (1.78)
Processed meat (SVs/d), mean (SD)	1.37 (1.34)	1.54 (1.38)	1.7 (1.47)	1.86 (1.62)
Urinary sodium excretion, mean (SD), mg/24 h	4075.52 (1224.12)	4177.61 (1251.93)	4220.79 (1272.24)	4351.5 (1328.88)
Urinary potassium excretion, mean (SD), mg/24 h	2806.43 (660.18)	2764.41 (663.78)	2730.87 (665.2)	2678.48 (665.63)
Hypertension, n (%)	152,444 (56.19)	74,621 (54.56)	31,193 (55.17)	12,895 (54.69)
Diabetes, n (%)	15,820 (5.83)	8511 (6.22)	3503 (6.2)	1568 (6.65)
Atrial fibrillation, n (%)	3994 (1.47)	1942 (1.42)	833 (1.47)	338 (1.43)
Congestive heart failure, n (%)	277 (0.1)	162 (0.12)	72 (0.13)	41 (0.17)
Stroke, n (%)	4651 (1.71)	2240 (1.64)	1004 (1.78)	512 (2.17)
Asthma, n (%)	31,831 (11.73)	16,873 (12.34)	6972 (12.33)	3217 (13.64)

BMI, Body mass index; IQR, interquartile range; SD, standard deviation; SVs/d, servings/day

Table 2 shows the associations between the frequency of adding salt to foods and incident sleep apnea. During a median follow-up of 12.3 years (maximum follow-up of 15.6 years), we recorded 6394 incident sleep apnea events. Participants who had the habit of adding salt to foods were at higher risk for incident sleep apnea. The adjusted incidence rates for never/rarely, sometimes, usually and always adding salt to foods were 0.93 (95% CI, 0.90 to 0.96) cases, 1.15 (95% CI, 1.10 to 1.20) cases, 1.31 (95% CI, 1.22 to 1.39) cases and 1.60 (95% CI, 1.46 to 1.75) cases per 1000 person-years, respectively. In Cox proportional hazards regression model, after adjustment for age, sex, and race, participants who always added salt to foods had a 63% higher risk of incident sleep apnea (HR, 1.63; 95% CI, 1.48 to 1.80), compared with those never/rarely added salt to foods. In the multivariable-adjusted model, the HRs associated with sometimes, usually, and always adding salt to foods were 1.11 (95% CI, 1.04 to 1.17), 1.15 (95% CI, 1.07 to 1.24), and 1.24 (95% CI, 1.12 to 1.37), respectively.

**Table 2:** Cox proportional hazard model for the association between the frequency of adding salt to food and incident sleep apnea among 488,196 participants in UK biobank

	Frequency of adding salt to food				*p* for trend
	Never/rarely	Sometimes	Usually	Always	
No. of SA cases/PY	3,106/3,344,599	1,926/1,682,245	904/692,201	458/286,312	
Incident rate per 1000 PY (95% CI)	0.93 (0.90, 0.96)	1.15 (1.10, 1.20)	1.31 (1.22, 1.39)	1.60 (1.46, 1.75)	
Model 1	1 (ref)	1.21 (1.14, 1.28)	1.32 (1.23, 1.42)	1.63 (1.48, 1.80)	< 0.001
Model 2	1 (ref)	1.18 (1.11, 1.25)	1.27 (1.18, 1.37)	1.37 (1.24, 1.51)	< 0.001
Model 3	1 (ref)	1.10 (1.04, 1.16)	1.13 (1.05, 1.22)	1.21 (1.10, 1.34)	< 0.001
Model 4	1 (ref)	1.11 (1.04, 1.17)	1.15 (1.07, 1.24)	1.24 (1.12, 1.37)	< 0.001

Model 1: adjusted for sex, age, and race

Model 2: model 1 also adjusted for income, socioeconomic status, highest qualification, employment status, and assessment center

Model 3: model 2 also adjusted for smoking status, alcohol intake, physical activity, vegetable intake, fruits intake, red meats intake, processed meat intake, and body mass index

Model 4: model 3 also adjusted for multimorbidity, including diabetes, hypertension, atrial fibrillation, congestive heart failure, stroke, and asthma

PY, person-years; CI, confidence interval

Stratified analyses were conducted according to age, sex, race, Townsend deprivation index, physical activity, smoking status, moderate drinking, BMI, hypertension, diabetes, atrial fibrillation, congestive heart failure, and asthma (Table 3). The associations between the frequency of adding salt to foods and incident sleep apnea were significant among participants older than 60 years old, White, and those with lower Townsend deprivation index (all *P*-interaction < 0.01). In the sensitivity analyses, the associations between the habit of adding salt to foods and incident sleep apnea did not change appreciably: first, when we conducted a complete analysis; second, when we excluded participants with baseline cardiovascular diseases or cancer; third, when we treated the habit of adding salt to foods as a binary variable; fourth, when we excluded those with major dietary changes in the last five years; fifth, when we excluded those with OSA risk at baseline; sixth, when we further adjusted for menopause and history of hormone replacement therapy among female participants; seventh, when we further adjusted for other potential covariates including urine sodium and potassium, sleep duration, chronotype, weight change, drug uses. (Additional file 1: Tables S3, S4) Mediation analyses using the Cox and Hertzmark methods indicated that BMI's effect could explain 16.1% (95% CI, 9.1%-27.1%) of the impact of adding salt to food on incident sleep apnea and is highly significant with a *P* < 0.0001. Mediation effects were not detected for other candidates, including blood pressure, blood glucose, blood lipids, and C-reactive protein level.

**Table 3:** Cox proportional hazard model for the association between the frequency of adding salt to food and incident sleep apnea among 488,196 participants in UK biobank: subgroup analyses

Subgroups		N	Salt added to food				*P* for interaction
			Never/rarely	Sometimes	Usually	Always	
Age							
	< 60	277,282	1 (ref)	1.02 (0.95, 1.1)	1.01 (0.91, 1.12)	**1.17 (1.03, 1.33)**	0.002
	≥ 60	210,914	1 (ref)	**1.24 (1.13, 1.35)**	**1.35 (1.21, 1.51)**	**1.33 (1.13, 1.57)**	
Sex							
	Females	268,295	1 (ref)	1.04 (0.94, 1.15)	1.14 (0.99, 1.3)	**1.22 (1.03, 1.44)**	0.47
	Males	219,901	1 (ref)	**1.15 (1.07, 1.23)**	**1.17 (1.07, 1.28)**	**1.24 (1.09, 1.41)**	
Race							
	White	460,225	1 (ref)	**1.12 (1.06, 1.19)**	**1.17 (1.08, 1.26)**	**1.29 (1.16, 1.43)**	0.01
	Non-white	27,971	1 (ref)	0.95 (0.77, 1.16)	0.99 (0.76, 1.31)	0.9 (0.66, 1.23)	
Townsend deprivation index							
	< Median	243,789	1 (ref)	**1.18 (1.07, 1.29)**	**1.29 (1.15, 1.45)**	**1.32 (1.1, 1.58)**	0.003
	≥ Median	243,803	1 (ref)	1.06 (0.99, 1.14)	1.07 (0.97, 1.18)	**1.21 (1.07, 1.37)**	
Physical activity							
	Active	247,350	1 (ref)	**1.14 (1.04, 1.25)**	1.1 (0.98, 1.25)	**1.29 (1.09, 1.52)**	0.30
	Insufficient	133,972	1 (ref)	1.07 (0.96, 1.19)	**1.21 (1.05, 1.38)**	1.17 (0.95, 1.44)	
	Inactive	75,333	1 (ref)	1.1 (0.97, 1.24)	1.13 (0.96, 1.31)	**1.38 (1.15, 1.65)**	
Smoking status							
	Never	267,618	1 (ref)	**1.11 (1.02, 1.21)**	**1.16 (1.02, 1.3)**	**1.2 (1.01, 1.42)**	0.08
	Ever	167,709	1 (ref)	1.08 (0.98, 1.18)	**1.21 (1.08, 1.35)**	**1.36 (1.17, 1.59)**	
	Current	51,036	1 (ref)	**1.21 (1.03, 1.41)**	1.04 (0.85, 1.26)	1.23 (0.98, 1.53)	
Moderate drinking							
	Yes	256,789		1.05 (0.97, 1.14)	**1.13 (1.02, 1.26)**	**1.25 (1.06, 1.46)**	0.26
	No	149,500	1 (ref)	**1.14 (1.03, 1.26)**	1.12 (0.98, 1.28)	**1.19 (1.01, 1.4)**	
			1 (ref)				
BMI							
	< 25	162,199	1 (ref)	**1.25 (1.04, 1.51)**	**1.38 (1.07, 1.77)**	1.28 (0.88, 1.84)	0.84
	25.0–29.9	206,844	1 (ref)	1.09 (0.98, 1.21)	1.08 (0.94, 1.24)	1.13 (0.93, 1.38)	
	≥ 30.0	116,577	1 (ref)	**1.1 (1.02, 1.18)**	**1.15 (1.04, 1.27)**	**1.28 (1.13, 1.45)**	
Hypertension							
	Yes	271,153	1 (ref)	**1.12 (1.04, 1.19)**	**1.14 (1.04, 1.25)**	**1.16 (1.03, 1.31)**	0.39
	No	217,043	1 (ref)	1.08 (0.97, 1.21)	**1.18 (1.02, 1.36)**	**1.43 (1.19, 1.71)**	
Diabetes							
	Yes	29,402	1 (ref)	**1.17 (1.02, 1.34)**	1.19 (0.99, 1.43)	1.04 (0.80, 1.36)	0.44
	No	458,794	1 (ref)	**1.1 (1.03, 1.17)**	**1.15 (1.06, 1.25)**	**1.28 (1.15, 1.43)**	
Atrial fibrillation							
	Yes	7107	1 (ref)	0.89 (0.66, 1.2)	1.17 (0.79, 1.71)	1.08 (0.63, 1.85)	0.50
	No	481,089	1 (ref)	**1.11 (1.05, 1.18)**	**1.14 (1.06, 1.23)**	**1.23 (1.11, 1.37)**	
Congestive heart failure							
	Yes	552	1 (ref)	0.3 (0.08, 1.14)	0.18 (0.02, 2)	1.56 (0.3, 8.03)	0.12
	No	487,644	1 (ref)	**1.11 (1.05, 1.18)**	**1.16 (1.08, 1.25)**	**1.24 (1.12, 1.37)**	
Stroke							
	Yes	8407	1 (ref)	1.14 (0.84, 1.56)	1.41 (0.97, 2.06)	1.45 (0.87, 2.4)	0.94
	No	479,789	1 (ref)	**1.11 (1.04, 1.17)**	**1.15 (1.06, 1.24)**	**1.24 (1.12, 1.37)**	
Asthma							
	Yes	58,893	1 (ref)	1.03 (0.9, 1.16)	1.04 (0.87, 1.23)	1.14 (0.92, 1.42)	0.08
	No	429,303	1 (ref)	**1.13 (1.06, 1.21)**	**1.18 (1.09, 1.29)**	**1.27 (1.13, 1.42)**	

Model adjusted for sex, age, race, income, socioeconomic status, highest qualification, employment status, assessment center, smoking status, alcohol intake, physical activity, vegetable intake, fruits intake, red meats intake, processed meat intake, BMI, and multimorbidity (diabetes, hypertension, atrial fibrillation, congestive heart failure, stroke, and asthma)

Bold values mean* P* < 0.05

BMI, body mass index

## Discussion

In this large prospective study, the habit of adding salt to foods was associated with a higher risk of incident sleep apnea. Such associations were independent of recognized risk factors of sleep apnea, including sociodemographic factors, lifestyles, and multimorbidity.

Recently, in the sleep apnea treating field, high dietary salt has gained increasing interest by its potential role in the pathogenesis of sleep apnea. In this study, we discussed the relationship between salt (sodium) intake and sleep apnea from the perspective of the habit of adding salt to foods. To the best of our knowledge, this is the first large prospective study to examine the association between the habit of adding salt to foods and sleep apnea among the general population. Supporting the findings of our study, most studies revealed that high dietary salt intake was independently related to the presence of sleep apnea in hypervolemic conditions, such as heart failure [30], resistant hypertension [31], and hyperaldosteronism [31]. Previous studies indicated that modulation of sodium intake might be a therapeutic strategy to alleviate sleep apnea in participants with the disease in hypervolemic conditions. Interestingly, the best cutoff value of sodium intake that predicted the presence of sleep apnea in patients with heart failure was similar to that recommended for healthy adults that is, 2.3 g/day [30]. Moreover, a randomized controlled parallel study demonstrated reductions in the apnea–hypopnea index of about 22.3% using the low-salt diet in men with severe obstructive sleep apnea (OSA) [15]. Combining our findings led us to the implication that restriction of salt intake might exert a potential approach for sleep apnea prevention in the general population.

In 2003, the UK government developed a program of voluntary salt reduction, which led to a 15% reduction in the average salt intake of the population during the following 7 years [32]. Nevertheless, according to the National Diet and Nutrition Survey, no significant changes in salt intake between 2014 and 2018/19 in the UK were reported. [33] In 2018/2019, the arithmetic mean estimated salt intake (8.4 g/day) for adults aged 19 to 64 years was still 40% higher than the government-recommended maximum of 6 g/day [33]. There is a long way to go in salt reduction work. After a successful salt reduction program, it is also necessary to assess the change of incident sleep apnea.

The underlying mechanisms and causative pathways remain unclear. High sodium intake and renal dysfunction likely contributed to sleep apnea severity at least partially through fluid retention and increased overnight rostral fluid displacement [30, 31]. A previous study indicated that high sodium intake and renal dysfunction contribute to the pathogenesis of central sleep apnea (CSA) and OSA in heart failure patients [30]. Further experimental studies are needed to evaluate the cellular and molecular mechanism, which may provide novel preventive and therapeutic insights.

### Strengths and limitations of this study

This study has several major strengths, including the large sample size and the wealth of information, enabling us to adjust possible covariates and conduct comprehensive sensitivity and subgroup analyses. The following inevitable limitations deserve mentioning. First, it is challenging to distinguish subtypes of sleep apnea, including CSA and OSA, in this study. However, in reality, given considerable overlap between these two conditions, the more general term sleep apnea to encompass both subtypes’ manifestations of disordered ventilatory control might be more appropriate [34]. Second, the frequency of adding salt to foods is partly related to the salt intake of individuals. We conducted further adjustments for the estimated 24-h excretion of sodium and potassium in sensitivity analyses, and the results were not substantially changed. Third, this study was also limited by a single assessment of adding salt to foods at baseline. Major dietary changes may accompany by the change of habits of adding salt to foods. Therefore, we conducted a sensitivity analysis to exclude participants with major dietary changes in the last 5 years. Fourth, sleep apnea is a highly underestimated disease due to the low rate of diagnosis. To minimize the influence of undiagnosed cases of sleep apnea, we restricted our analyses to those participants with low OSA risk in the sensitivity analysis. Fifth, although we conducted the sensitivity analyses, the reverse causality between the habit of adding salt to foods and incident sleep apnea cannot be eliminated due to the nature of the observational study.

## Conclusions

The habit of adding salt to foods was independently related to higher risks of sleep apnea. Our findings support the benefits of a salt reduction program in preventing sleep apnea in the general population.

## Supplementary Information

**Additional file 1.** **Supplementary Table 1.** Illnesses definitions in UK Biobank study. **Supplementary Table 2.** Covariate definitions and methods of assessment. **Supplementary Table 3.** Cox proportional hazard model for the association between the habit of adding salt to food (binary variable) and incident sleep apnea among participants in UK biobank (n = 488,196). **Supplementary Table 4.** Sensitivity analyses for association of the frequency of adding salt to foods with incident sleep apnoea.

## Data Availability

The data that support the findings of this study are available from the UK Biobank.

## Abbreviations

ICD: International classification of disease
BMI: Body mass index
HR: Hazard ratio
CI: Confidence interval
CSA: Central sleep apnea
OSA: Obstructive sleep apnea
