# Supplementary material for: Eating habit of adding salt to foods and incident sleep apnea: a prospective cohort study
Source: Respir Res. 2023 Jan 7;24:5. doi: 10.1186/s12931-022-02300-6 (PMC9826571; doi:10.1186/s12931-022-02300-6)
Supplement: Supplementary file 1 — Additional file 1. Supplementary Table 1. Illnesses definitions in UK Biobank study. Supplementary Table 2. Covariate definitions and methods of assessment. Supplementary Table 3. Cox proportional hazard model for the association between the habit of adding salt to food (binary variable) and incident sleep apnea among participants in UK biobank (n = 488,196). Supplementary Table 4. Sensitivity analyses for association of the frequency of adding salt to foods with incident sleep apnoea. [file 12931_2022_2300_MOESM1_ESM.docx]

**Supplementary Table 1.** Illnesses definitions in UK Biobank study

**Supplementary Table 2.** Covariate definitions and methods of assessment

**Supplementary Table 3.** Cox proportional hazard model for the association between the habit of adding salt to food (binary variable) and incident sleep apnea among participants in UK biobank (n=488,196)

**Supplementary Table 4.** Sensitivity analyses for association of the frequency of adding salt to foods with incident sleep apnoea

| **Supplementary Table 1. Sleep apnoea definition in UK Biobank study** | | | | | | |
| --- | --- | --- | --- | --- | --- | --- |
|  | **Hospital inpatient records** | | **Death registries** | | **Self-reported fields** | **Primary care** |
|  | **ICD-9** | **ICD-10** | **ICD-9** | **ICD-10** |  |  |
| **Code** | - | G47.3 | - | G47.3 | 1123 | Fy03., Fy04., H5B.., H5B0., R0051, R0053, X0083, X0084, X0085, X0086, X0087, X00pU, X20L9, X20M9, XE2nU, XM0Go, XaEGP, XaQwj, .H66., .R051, .R053, 38Da., X008F, X76Gw, X76Hk, XM08E |
| **Number of cases** | 10108 | | 107 | | 1862 | 3627 |
| Abbreviations: ICD, International Classification of Diseases. | | | | | | |

| **Supplementary Table 2. Covariate definitions and method of assessment** | | | |
| --- | --- | --- | --- |
| **Covariate** | **Description** | **Assessment** | **UK Biobank Code** |
| Age (years) | Age in years | Date attended baseline assessment minus date of birth | 21003 |
| Sex | Male, female | NHS derived and/or touchscreen questionnaire | 31 |
| Ethnicity | White, non-White (Mixed, Asian, Black, Chinese, Other) | Touchscreen questionnaire: “What is your ethnic group?” | 21000 |
| Country | England, Wales, Scotland | Location of baseline assessment center attended | 54 |
| Education | Higher (college or university degree, other professional qualifications), upper secondary (A levels, AS levels or equivalent), lower secondary (O levels/GCSEs or equivalent, CSEs or equivalent), vocational (NVQ or HND or HNC or equivalent); no secondary education (none of the above) | Touchscreen questionnaire: “Which of the following qualifications do you have?” | 6138 |
| Employment | Employed, Unemployed, retired | Employment status was ascertained by touchscreen and verbal interview. | 6142, 20119 |
| Household income | <£18 000, £18 000-£30 999, £31 000-£51 999, £52 000-£100 000, >£100 000 | Touchscreen question "What is the average total income before tax received by your HOUSEHOLD?" | 738 |
| Socioeconomic status | Quartiles | Townsend deprivation index calculated prior to participant joining UK Biobank. Based on the preceding national census output areas. Each participant is assigned a score corresponding to the output area in which their postcode is located | 189 |
| Smoking status | Never, previous, current | A series of questions about smoking in touchscreen questionnaire: “Do you smoke tobacco now?”, “In the past, how often have you smoked tobacco?”, “Have you tried to give up smoking?” | 20116 |
| Alcohol intake | Moderate alcohol intake (yes/no) | Moderate alcohol intake was defined as <40 g/day for men; <24 g/day for women. | 1558, 1568, 1578, 1588, 1598, 1608, 5364, 4407, 4418, 4429, 4440, 4451, 4462 |
| Vegetables intake | Quartiles | Touchscreen questionnaire: “On average how many heaped tablespoons of COOKED vegetables would you eat per DAY?”, “On average how many heaped tablespoons of SALAD or RAW vegetables would you eat per DAY?”  Vegetables intake was defined as the total intake of cooked and raw vegetables per day. | 1289, 1299 |
| Fruit intake | Quartiles | Touchscreen questionnaire: “About how many pieces of FRESH fruit would you eat per DAY?”, “About how many pieces of DRIED fruit would you eat per DAY?”  Fruit intake was defined as the total intake of fresh and dried fruit per day. | 1309, 1319 |
| Processed meat intake | Quartiles | Touchscreen questionnaire: “How often do you eat processed meats?” | 1349 |
| Unprocessed red meat intake | Quartiles | Touchscreen questionnaire: “How often do you eat beef/lamb/mutton/pork?” | 1369, 1379, 1389 |
| Physical activity | Inactive, insufficient, and active | The information on frequency and duration of moderate and vigorous physical activities were collected by touchscreen questionnaire. | 884, 894, 904,914 |
| BMI (kg/m^2^) | Underweight or normal (<25), overweight (25-29) or obese (≥30) | Physical examination: body mass index | 21001 |
| Hypertension | No, yes | Hypertension cases were ascertained from hospital inpatient records, death registries, self-reported fields and Office of Population Censuses and Surveys Classification of Interventions and Procedures, version 4 (OPCS-4). | 41270, 41271, 40001, 40002, 20002, 41272 |
| Diabetes | No, yes | Diabetes cases were ascertained from hospital inpatient records, death registries, self-reported fields and Office of Population Censuses and Surveys Classification of Interventions and Procedures, version 4 (OPCS-4). | 41270, 41271, 40001, 40002, 20002, 41272 |
| Atrial fibrillation | No, yes | Atrial fibrillation cases were ascertained from hospital inpatient records, death registries, self-reported fields and Office of Population Censuses and Surveys Classification of Interventions and Procedures, version 4 (OPCS-4). | 41270, 41271, 40001, 40002, 20002, 41272 |
| Congestive heart failure | No, yes | Congestive heart failure cases were ascertained from hospital inpatient records, death registries, self-reported fields and OPCS-4. | 41270, 41271, 40001, 40002, 20002, 41272 |
| Stroke | No, yes | Stroke cases were ascertained from hospital inpatient records, death registries, self-reported fields and OPCS-4. | 41270, 41271, 40001, 40002, 20002, 41272 |
| Asthma | No, yes | Asthma cases were ascertained from hospital inpatient records, death registries, and self-reported fields. | 41270, 41271, 40001, 40002, 20002, 6152 |
| Drugs use (opiates, benzodiazepines and other depressants) | No, yes | Drugs use (opiates, benzodiazepines and other depressants) was ascertained by verbal interview. | Field id-20003:  Code-1140921600 1141180212 1141190158 1140867888 1140882236 1140879540 1140867876 1140879544 1140867860 1140867878 1141200564 1141201834 1140916282 1140867938 1140867948 1140879616 1140879620 1140867690 1140909806 1140867624 1140867628 1140867640 1140882312 1140879630 1140867712 1140867726 1140882310 1140867818 1140867756 1140867758 1140867784 1140879556 1140867812 1141152732 1140867856 1140867850 1140867852 1140867914 1140910820 1140867916 1140867920 1140867922 1140867944 1140917460 1140879634 1141151978 1141151982 1141199446 1141176854 |
| Menopause | No, yes, NA (male) | Touchscreen questionnaire: "Have you had your menopause (periods stopped)?" | 2724 |
| Hormone-replacement therapy | No, yes, NA (male) | Touchscreen questionnaire: "Have you ever used hormone replacement therapy (HRT)?" | 2814 |
| Sodium in urine | Quartiles | Measured by ISE (ion selective electrode) analysis using Beckman Coulter AU5400. | 30530 |
| Potassium in urine | Quartiles | Measured by ISE (ion selective electrode) analysis on a Beckman Coulter AU5400. | 30520 |
| Sleep duration | <7, 7~8, >9, hours/day | Touchscreen question "About how many hours sleep do you get in every 24 hours? (Please include naps)" | 1160 |
| Morning/evening person (chronotype) | 'morning' person, 'evening' person | Touchscreen question "Do you consider yourself to be?" | 1180 |
| Major dietary changes in the last 5 years | No, yes | Touchscreen question "Have you made any major changes to your diet in the last 5 years?" | 1538 |
| Weight change compared with 1 year ago | No - weigh about the same  Yes - gained weight  Yes – lost weight  Unknown or missing | Touchscreen question "Compared with one year ago, has your weight changed?" | 2306 |
| Waist circumference | Continuous, cm | Physical examination: Waist circumference | 48 |
| Drugs for blood pressure and blood sugar | No, yes | Touchscreen question "Do you regularly take any of the following medications? (you can select more than one answer)"  -With the answer of “Blood pressure medication” and “Insulin”. | 6177 |

| **sTable 3. Cox proportional hazard model for the association between the habit of adding salt to food (binary variable) and incident sleep apnea among participants in UK biobank (n=488,196)** | | |
| --- | --- | --- |
|  | **Hazard ratio (95% confident interval)** | |
|  | **Never/rarely do not generally add salt at the table** | **Generally add salt at the table** |
| No. of SA cases/PY | 5,032 / 5,026,844 | 1,362 / 978,513 |
| Incident rate per 1,000 PY (95% CI) | 1.001 (0.974, 1.029) | 1.392 (1.320, 1.468) |
| Model 1 | 1 (ref.) | 1.32 (1.24, 1.40) |
| Model 2 | 1 (ref.) | 1.22 (1.15, 1.30) |
| Model 3 | 1 (ref.) | 1.12 (1.05, 1.19) |
| Model 4 | 1 (ref.) | 1.13 (1.06, 1.20) |
| Model 1: adjusted for sex, age, and race Model 2: model 1 also adjusted for income, socioeconomic status, highest qualification, employment status, and assessment center Model 3: model 2 also adjusted for smoking status, alcohol intake, physical activity, vegetable intake, fruits intake, red meats intake, processed meat intake, and body mass index Model 4: model 3 also adjusted for multimorbidity, including diabetes, hypertension, atrial fibrillation, congestive heart failure, stroke, and asthma. PY, person-years; CI, confidence interval. | | |

| **sTable 4. Sensitivity analyses for association of the frequency of adding salt to foods with incident sleep apnoea** | | | | | |
| --- | --- | --- | --- | --- | --- |
|  | **N** | **Frequency of Adding Salt to Foods** | | | |
|  |  | **Never/rarely** | **Sometimes** | **Usually** | **Always** |
| Main analysis | 488,196 | 1 (ref) | 1.11 (1.04, 1.17) | 1.15 (1.07, 1.24) | 1.24 (1.12, 1.37) |
| Sensitivity analysis 1 | 325,556 | 1 (ref) | 1.08 (1.01, 1.16) | 1.12 (1.02, 1.23) | 1.26 (1.10, 1.43) |
| Sensitivity analysis 2 | 404,411 | 1 (ref) | 1.10 (1.03, 1.18) | 1.16 (1.06, 1.26) | 1.20 (1.06, 1.35) |
| Sensitivity analysis 3 | 298,127 | 1 (ref) | 1.03 (0.95, 1.13) | 1.12 (1.002, 1.24) | 1.15 (0.995, 1.33) |
| Sensitivity analysis 4 | 366,660 | 1 (ref) | 1.04 (0.95, 1.14) | 1.20 (1.07, 1.35) | 1.18 (0.998, 1.39) |
| Sensitivity analysis 5 | 268,295 | 1 (ref) | 1.04 (0.94, 1.14) | 1.13 (0.99, 1.29) | 1.21 (1.02, 1.43) |
| Sensitivity analysis 6 | 488,196 | 1 (ref) | 1.11 (1.04, 1.17) | 1.14 (1.05, 1.23) | 1.25 (1.13, 1.39) |
| Sensitivity analysis 7 | 488,196 | 1 (ref) | 1.10 (1.04, 1.17) | 1.15 (1.06, 1.24) | 1.21 (1.10, 1.34) |
| Sensitivity analysis 8 | 488,196 | 1 (ref) | 1.10 (1.04, 1.17) | 1.15 (1.06, 1.24) | 1.23 (1.11, 1.36) |
| Sensitivity analysis 9 | 488,196 | 1 (ref) | 1.11 (1.05, 1.17) | 1.15 (1.06, 1.24) | 1.22 (1.1, 1.36) |
| Sensitivity analysis 10 | 488,196 | 1 (ref) | 1.09 (1.03, 1.16) | 1.11 (1.03, 1.2) | 1.18 (1.06, 1.3) |
| Sensitivity analysis 11 | 488,196 | 1 (ref) | 1.10 (1.04, 1.17) | 1.14 (1.06, 1.23) | 1.21 (1.09, 1.34) |
| Sensitivity analysis 12 | 488,196 | 1 (ref) | 1.12 (1.06, 1.19) | 1.17 (1.09, 1.26) | 1.26 (1.14, 1.39) |
| Sensitivity analysis 13 | 488,196 | 1 (ref) | 1.11 (1.05, 1.18) | 1.15 (1.07, 1.24) | 1.23 (1.11, 1.37) |
| Main analysis: Adjusted for sex, age, race, income, socioeconomic status, highest qualification, employment status, assessment center, smoking status, alcohol intake, physical activity, vegetable intake, fruits intake, red meats intake, processed meat intake, BMI, and multimorbidity (diabetes, hypertension, atrial fibrillation, congestive heart failure, stroke, and asthma). | | | | | |
| Sensitivity analysis 1: Complete case analysis (excluding any missing data). Sensitivity analysis 2: Excluding participants with baseline CVDs or cancer. Sensitivity analysis 3: Excluding participants with major dietary changes in the last five years. Sensitivity analysis 4: Excluding participants with obstructive sleep apnoea risk at baseline. Sensitivity analysis 5: Further adjusted for menopausal status and hormone replacement therapy among females. Sensitivity analysis 6: Further adjusted for sodium and potassium in urine (quintiles). Sensitivity analysis 7: Further adjusted for sleep duration. Sensitivity analysis 8: Further adjusted for chronotype. Sensitivity analysis 9: Further adjusted for weight change compared with 1 year ago.  Sensitivity analysis 10: Further adjusted for waist circumference.  Sensitivity analysis 11: Further adjusted for depressant use. Sensitivity analysis 12: Further adjusted for drugs for blood pressure, and blood sugar.  Sensitivity analysis 12: Further adjusted for drugs for diuretics. | | | | | |
